# Physician turnover in primary health care services in the East Zone of São Paulo City, Brazil: incidence and associated factors

**DOI:** 10.1186/s12913-022-07517-1

**Published:** 2022-02-04

**Authors:** Monique M. M. Bourget, Alex J. F. Cassenote, Mário C. Scheffer

**Affiliations:** 1 Program of Collective Health, Faculty of Medicine of the São Paulo University (FMUSP), São Paulo, SP Brazil; 2 Department of Gastroenterology, Faculty of Medicine of the São Paulo University (FMUSP), São Paulo, SP Brazil; 3 Brazilian Medical Demography Research Group, Faculty of Medicine of the São Paulo University (FMUSP), São Paulo, SP Brazil; 4 Department of Preventative Medicine, Faculty of Medicine of the São Paulo University (FMUSP), São Paulo, SP Brazil; 5 Evidence Based Medicine Discipline, Santa Marcelina Faculty, São Paulo, SP Brazil

**Keywords:** Employment, Physicians, Primary health care, Personnel turnover, Health centers, Survival analysis

## Abstract

**Background:**

The shortage and high turnover of physicians is a recurrent problem in health care systems; this is especially harmful to the expansion and full operation of primary health care (PHC). The aim of this paper is to analyze incidence and associated factors with physician turnover in primary health care services in the East Zone of São Paulo City.

**Methods:**

This is a retrospective cohort study of 1378 physicians over a 15 years’ time period based on physicians’ administrative records from two distinct secondary databases. Physicians’ individual characteristics were analyzed including graduation and specialization. Survival analysis techniques such Kaplan-Meier and Cox Regression were used to analyze the termination of contract.

**Results:**

One thousand three hundred seventy-eight physicians were included in the study of which 130 [9.4%(CI95 8.0–11.1%)] remained in the PHC services. The mean and median time until the occurrence of the physician leaving the service was 2.14 years (CI95% 1.98–2.29 years) and 1.17 years [(CI95% 1.05–1.28 years)]. The probability of contract interruption was 45% in the first year and 68% in the second year. Independent factors associated with TEC were identified: workload of 40 h/week HR = 1.71 [(CI95% 1.4–2.09), *p* < 0.001]; initial salary ≤1052 BGI HR = 1.87 [(CI95 1.64–2.15), *p* < 0.001]; time since graduation ≤2 years HR =1.36 [(CI95 1.18–1.56), *p* < 0.001]; and the conclusion of residency in up to 3 years after leaving the service HR = 1.69 [(CI95 1.40–2.04), *p* < 0.001].

**Conclusions:**

The time of employment of the physician in PHC was relatively short, with a high probability of TEC in the first year. Modifiable factors such as working hours, starting salary, time since graduation from medical school and need to enter in a residency program were associated with TEC. In pointing out that modifiable factors are responsible for long term employment or the end of contract of physicians in PHC services of the Unified Health System in the periphery of a metropolitan area, the study provides support for the planning, implementation and management of policies and strategies aimed at attracting and retaining physicians in suburban, priority or underserved regions.

**Supplementary Information:**

The online version contains supplementary material available at 10.1186/s12913-022-07517-1.

## Background

Health professionals are the cornerstone of health care systems and contribute to the development of nations, representing more than 10% of total employment in many countries [1–3]. The composition of the workforce in health and the mechanisms to guarantee financial sustainability has mobilized international organizations and leaders in health care all over the world. The great challenge is to have a workforce that is qualified, sufficient, permanent and adequate to each environment [4–8].

Workforce depends on many factors like the political system of the country, national policy; educational system, quality of training; country’s economy and financing of the educational and health care system; waging policies, working conditions, distribution and insertion of professionals in the system. Additional topics include epidemiology and demographics, legislation, practice regulations and professional councils and popular councils, covenant and agreement between parties [9–14]. Worldwide, half the population lives in poor areas but only 23% of health professionals are located in these same poor areas, hence, there are many care gaps [15–19]. This study will try to identify the factors that could help retain the profissionals.

The working place of a physician is a complex and multifactorial choice. Some of the issues are lack of attractiveness for living and working conditions, forms of employment, contracts and remuneration of professionals, lower prestige and professional status in relation to primary care medical activities and specialties, lower attraction to more remote regions which may be perceived to have less educational, cultural and demographic opportunities; in addition, less health resources and high disease burden make provision of health care more challenging [20–23].

PHC is a key element to the constitution of national health care systems, with the capacity to impact positively on health indicators and with a great potential to assist in the optimal use of resources and organize the network. PHC has become responsible for the organization of health care of individuals, families and the population over time, leading to better health indicators, improved efficiency and lower costs to the system [24–26].

In Brazil, The Family Health Strategy (FHS) is a strategic program of the Brazilian Unified Health System (UHS) that initiated in 1994 and over the last 25 years has rapidly expanded. This policy had a positive impact on population’s access to health services, leading to a massive growth in the number of medical consultations per capita between 1990 and 2009; there has been a remarkable diminution of over 70% of infant mortality and of hospitalization for strokes and acute respiratory infections [27–30]. However, one of the big challenges of PHC has been the high turnover and subsequent lack of medical workforce, which resulted in the inability to further expand the FHS in many locations [31–33].

The difficulties of attracting and retaining medical professionals, especially in PHC, in Brazil and in other countries, have been the subject of several studies [16, 34–39]. Similar to other countries [40, 41], doctors in Brazil have multiple jobs, great mobility throughout their professional career and different possibilities of insertion in a segmented health system where the public and private sectors are in constant interaction and overlapping [42–44]. More than 50% of Brazilian physicians provide care in both public and private sectors [45] which may contribute to reducing the supply of doctors and service in the public sector; this can potentially adversely impact on the expansion capacity of PHC, accessibility and quality of care in public health care system [26]. The aim of this paper is to analyze incidence and associated factors with physician’s turnover in primary health care services in East Zone of São Paulo City which adopted a public-private partnership with many institutions since 2001, and Santa Marcelina Institution was the first one to sign such a contract of the management of the health care units. A study of this magnitude was unprecedented in Brazil, and also intended to contribute to the gap of individual factors that impact the turnover of professionals in the service in which they are inserted.

## Methods

### Study design, research scenario and ethical consideration

**Fig. 1:**
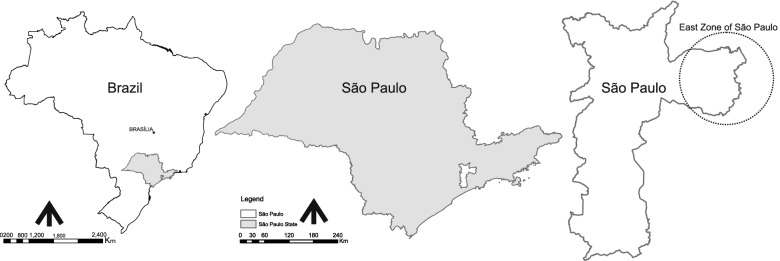
Study location. Dotted circle shows East Zone of São Paulo City

This is a retrospective longitudinal cohort study without a control group developed in the East Zone of São Paulo City, Brazil (Fig. 1).

The East Zone has great relevance to municipal health because of its high population density: in the last census of 2010, about 3,998,237 million people live in the region (35.5% of the total population of the capital - 11,253,503) [46]. With a large geographical extension, it concentrates the lowest per capita income of the municipality, has a high poverty rate and health problems common to the most peripheral regions and of high economic and social vulnerability. Most districts in the east have a human development index-HDI below the municipality and state average [47]. 70% of all health units are sob a contract managed by the Santa Marcelina institution (117 units of which 56 operate with FHS teams).

### Databases, inclusion criteria and ethical considerations

The main database was provided by the Santa Marcelina Human Resources Management Department and included information on the functional and administrative registration of physicians who have worked at PHC services administered by the Institution between 2001 and 2016. All variables that carry labor information originated from this database.

The second database was obtained from the study on Medical Demography In Brazil, which included demographic data from all physicians registered at the Medical Regional Councils (CRMs), as well as data from the National Commission of Medical Residency (CNRM) and all the Medical Specialty Societies associated to the Brazilian Medical Association (AMB).

All 1378 physicians admitted between January 1rst of 2001 and December 31rst of 2016 who worked or were still working in PHC in the East Zone of the city of São Paulo, Brazil, in a Traditional Health Care Unit or a Unit operating the Family Health Strategy, were included. This paper was performed in accordance with STROBE Statement for cohort studies.

The research followed all ethical parameters required by the Resolution N° 466/2012 of the National Research Ethics Commission of the National Health Council and was approved by the Research Ethical Committee of the Medical Faculty of São Paulo University (#66147417.0.0000.0065). Prior to the start this study, written consent and the required administrative permissions were obtained from the Santa Marcelina authorities to access human resource data. The IRB waived the use of the informed consent because the data accessed did not contain information that would allow personal identification. Confidentiality and privacy of all physician’s data was ensured, used solely and exclusively as a whole for statistical purposes.

### Variables and outcome definitions

After standardization of nomenclatures and typing in the final database, 15 variables were defined for study: work time, termination of contract (TEC), gender, age, year of initial employment date, weekly workload, place of work, initial salary at employment date, time frame since graduation at the time of initial employment, city of residency, type of graduation school, city of graduation, specialty on initial employment date, specialty at the present moment and medical residency after employment. The definitions and details of the variables are described in detail in the supplementary material.

### Data analysis

Descriptive statistics including absolute and relative frequencies and 95% confidence intervals (95%CI) were used. Estimates of the average time until the occurrence of the TEC, including a 95% confidence interval (95% CI) were calculated using Kaplan-Meier and for testing the hypothesis that the different factors had no impact on “survival”, a log rank statistic was used.

Cox regression was used to evaluate the multiple influence of the factors on the time until the occurrence of the termination of the contract in PHC. Backward selection procedure was the selection method adopted for the regression. To evaluate the statistical significance of the model coefficient Wald statistic was used and to examine the proportional hazards assumption for a Cox regression we used graphical diagnostics based on the scaled Schoenfeld residuals from coxph package in R.

All tests considered a bidirectional α of 0.05 and a confidence interval (CI) of 95% and were performed with computational support from the software IBM SPSS 25 (Statistical Package for the Social Sciences), R-GUI version 3.6.2 (http://www.r-project.org/) Excel 2016® (Microsoft Office).

## Results

The profiles of the 1378 physicians enrolled between 2001 and 2016 are shown in Table 1. From the total of physicians, 725 [52,6%(CI95%50,0%-55,2%)] were female, the mean age at enrollment was 32,6 years (±9,25), the minimum age being 22 years and the maximum 78 years and only 130[9,4%(CI958.0–11.1%)] remained in the services of the PHC of the Santa Marcelina Institution until December 2016.

**Table 1:** Distribution of the physicians that are being analyzed in the study, according to the selected variables, including absolute frequency, relative frequency and confidence interval of 95% (CI 95%)

Variable	N	%
Gender		
*Female*	725	52.6%
*Male*	653	47.4%
Age		
*≤ 25 years*	269	19.5%
*25 --| 30 years*	492	35.7%
*≥ 30 years*	617	44.8%
The year of employment		
*< 2005*	217	15.7%
*2005 --| 2010*	526	38.2%
*2010 --| 2015*	499	36.2%
*≥ 2015*	136	9.9%
Working Hours		
*40 h/weekly*	1079	78.3%
*< 40 h/weekly*	299	21.7%
Modality of working^a^		
*ESF*	1222	88.7%
*UBS*	156	11.3%
Initial wage^b^		
*≤ 1.052 BGI*	684	49.6%
*> 1.052 BGI*	694	50.4%
Years since graduation		
*≤ 2 years*	743	53.9%
*> 2 years*	635	46.1%
City of origin		
*São Paulo*	1077	78.2%
*Others*	301	21.8%
School of graduation		
*Public*	336	28.1%
*Private*	858	71.9%
Local of graduation		
*City of São Paulo*	195	16.3%
*Other municipalities*	999	83.7%
Medical Specialty^c^		
*Non specialist*	1232	89.4%
*Specialist*	146	10.6%
Medical Residency^d^		
*No*	1099	88.1%
*Yes*	149	11.9%
Specialty at the end of study		
*Non specialist*	754	55.0%
*Specialist*	617	45.0%
TEC-TEC		
*Yes*	1248	90.6%
*No*	130	9.4%

The Big Mac Index (BMI) created in 1986, calculated by The Economist, is an index based on the price of the Big Mac sandwich in more than 100 countries to explain the economy concept of parity in the purchasing power

^a^Work modality in PHC: ESF FHS – Family Health Strategy and BCU – Basic Care Unit

^b^Monthly wage adjusted by BGI - Big Mac Index

^c^Medical specialty at the initial time of the contract

^d^Medical Residency concluded after a minimum of 3 years after the end of the contract

**Fig. 2:**
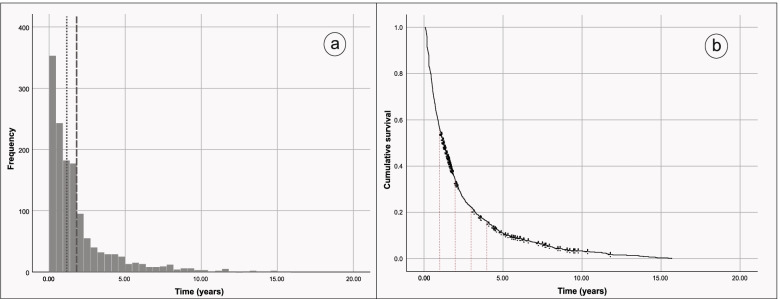
Characterization of follow-up time and cumulative survival in the set of physician enrolled patients. **a** the median is represented by the dotted line. The mean is represented by the dashed line. **b** dashed lines represent the years completed until a follow-up of 5 years

These physicians were followed for a total of 2.577,52 years. The median time of observation was of 1,87 years (±2,29 years) with a mean of 1,13 years (IIQ 0,45 a 2,17 years). A time distribution of the follow-up can be visualized in Fig. 2a. A density of the incidence of ending a contract of the service, was of 484,3 persons-year (CI95% 458,0-511,7).

The mean time until TEC was 2,14 years (CI95% 1,98–2,29 years) with a median of 1,17 years [(CI95% 1,05–1,28 years)] (Fig. 2a). The probability of ending the contract in the first year is 45%, increasing in the following years, with 68% in the second year, 78,5% in the third year and 84,2% in the fourth year, arriving to 89% of physicians that end their contract in the fifth year of follow- up (Fig. 2b).

Crude analysis showed that the following factors were significantly associated with TEC rate: age at initial employment, working hours, modality of unit, initial wage, time since graduation (years) and conclusion of the medical residency in up until 3 years after the end of the contract. The impact of each variable is described in detail in the Table 2.

**Table 2:** Estimates of the mean and median of the time frame until the occurrence of the end of the contract according with the different factors of the study including the confidence interval of 95% (CI 95%) and the descriptive level

	Total	End of contract	%	Mean (CI95%)	Median (CI95%)	***p***-value*
Gender						
*Female*	725	658	90.8%	2.1 (1.9–2.3)	1.1 (1.0–1.3)	0.529
*Male*	653	590	90.4%	2.2 (2.0–2.4)	1.2 (1.0–1.4)	
Age at initial employment						
*≤ 25 years*	269	248	92.2%	1.4 (1.2–1.6)	0.8 (0.6–1.0)	< 0.001
*25 --| 30 years*	492	456	92.7%	1.8 (1.6–2.1)	1.1 (0.9–1.3)	
*≥ 30 years*	617	544	88.2%	2.7 (2.4–3.0)	1.5 (1.3–1.7)	
Working Hours						
*40 h/weekly*	1079	999	92.6%	2.0 (1.9–2.2)	1.1 (1.0–1.2)	0.002
*< 40 h/weekly*	299	249	83.3%	2.6 (2.2–3.0)	1.5 (1.1–1.9)	
Modality of Unit						
*FSH*	1222	1117	91.4%	2.1 (1.9–2.2)	1.2 (1.0–1.3)	0.019
*TBCU*	156	131	84.0%	2.7 (2.1–3.2)	1.5 (1.0–1.9)	
Initial wage**						
*≤ 1.052 BGI*	684	612	89.4%	1.5 (1.4–1.7)	0.8 (0.7–0.9)	< 0.001
*> 1.052 BGI*	698	632	90.5%	2.6 (2.3–2.8)	1.5 (1.4–1.7)	
Time since graduation (years)						
*≤* 2 years	743	669	90.0%	1.7 (1.5–1.8)	1.0 (0.8–1.1)	< 0.001
> 2 years	635	579	91.2%	2.6 (2.4–2.9)	1.5 (1.3–1.7)	
City of residence						
*São Paulo*	1077	983	91.3%	2.1 (2.0–2.3)	1.2 (1.1–1.3)	0.975
*Others*	301	265	88.0%	2.1 (1.8–2.4)	1.1 (0.9–1.3)	
School of graduation						
*Public*	336	310	92.3%	2.1 (1.8–2.4)	1.0 (0.8–1.2)	0.852
*Private*	858	785	91.5%	2.0 (1.8–2.2)	1.1 (1.0–1.2)	
Local of graduation						
*São Paulo*	195	171	87.7%	1.9 (1.6–2.1)	1.3 (1–1.5)	0.990
*Other locals*	999	924	92.5%	2.0 (1.9–2.2)	1.1 (1–1.2)	
Specialty at the time of employment						
*Non specialist*	1232	1113	90.3%	2.1 (2.0–2.3)	1.1 (1.0–1.2)	0.199
*Specialist*	146	135	92.5%	2.3 (1.9–2.7)	1.5 (1.2–1.9)	
Medical Residency***						
*No*	1099	1099	100.0%	1.9 (1.8–2.0)	1.1 (1.0–1.2)	< 0.001
*Yes*	149	149	100.0%	0.8 (0.7–1.0)	0.7 (0.6–0.7)	
*General*	1371	1242	90.6%	2.1 (2.0–2.3)	1.2 (1.1–1.3)	

The variables of the year on contract and actual specialty were not analyzed based on criteria from the researchers

^*^ *p*-value calculated by the method of Log Rank (Mentel-Cox)

^**^Monthly wage adjusted according to Big Mac Index

^***^Conclusion of Medical Residency up until 3 years after the end of the contract

The adjusted analysis resulted in six distinct models (Table 3). All models present significative explanation (*p* < 0,001) in relation to the empty model. In the model VI, the best one, four variables remained statistically significant: a) working hours of 40 h/week HR = 1,71 [(CI95%1,4-2,09), *p* < 0,001]; b) initial wage ≤1.052 BGI HR =1,87 [(CI95 1,64–2,15), *p* < 0,001]; c) time since graduation ≤2 years HR =1,36 [(CI95 1,18-1,56), *p* < 0,001]; and d) conclusion of medical residency up until 3 years after the end of the contract HR =1,69 [(CI95 1,40-2,04), *p* < 0,001].

**Table 3:** Models used to study the time until the occurrence of the end of the contract including *hazard ratio* (HR) with a confidence interval of 95% (CI 95%) and descriptive level

	Model I		Model II		Model III		Model IV		Model V		Model VI	
	***p***-value*	HR (CI95%)	***p***-value*	HR (CI95%)	***p***-value*	HR (CI95%)	***p***-value*	HR (CI95%)	***p***-value*	HR (CI95%)	***p***-value*	HR (CI95%)
**Gender**												
*Male*		1.00		1.00								
*Female*	0.519	1.04 (0.92–1.18)	0.506	1.04 (0.92–1.18)	–	–	–	–	–	–	–	–
**Age at initial employment**												
*≤ 25 years*		1.00		1.00		1.00		1.00		1.00	–	–
*25 --| 30 years*	0.188	1.15 (0.94–1.40)	0.185	1.15 (0.94–1.40)	0.164	1.15 (0.94–1.41)	0.153	1.16 (0.95–1.42)	0.146	1.16 (0.95–1.42)	–	–
*≥ 30 years*	0.909	0.99 (0.85–1.16)	0.919	0.99 (0.85–1.16)	0.945	0.99 (0.85–1.16)	0.930	0.99 (0.85–1.16)	0.905	0.99 (0.85–1.16)	–	–
**Working hours**												
*< 40 h/week*		1.00		1.00		1.00		1.00		1.00		1.00
*40 h/week*	< 0.001	1.74 (1.42–2.13)	< 0.001	1.73 (1.41–2.12)	< 0.001	1.72 (1.41–2.10)	< 0.001	1.72 (1.40–2.10)	< 0.001	1.70 (1.39–2.08)	< 0.001	1.71 (1.4–2.09)
**Modality of Unit**												
*FSH*		1.00		1.00		1.00		1.00		1.00		1.00
*TCHU*	0.069	0.78 (0.60–1.02)	0.055	0.78 (0.60–1.01)	0.055	0.78 (0.60–1.01)	0.057	0.78 (0.60–1.01)	0.064	0.78 (0.61–1.02)	0.072	0.79 (0.61–1.02)
Initial wage**												
*> 1.052 BGI*		1.00		1.00		1.00		1.00		1.00		1.00
*≤ 1.052 BGI*	< 0.001	1.89 (1.65–2.17)	< 0.001	1.89 (1.65–2.17)	< 0.001	1.89 (1.65–2.16)	< 0.001	1.89 (1.65–2.16)	< 0.001	1.89 (1.65–2.16)	< 0.001	1.87 (1.64–2.15)
**Time since graduation (years)**												
> 2 years		1.00		1.00		1.00		1.00		1.00		1.00
*≤* 2 years	0.002	1.32 (1.11–1.57)	0.002	1.31 (1.11–1.55)	0.002	1.31 (1.11–1.55)	0.002	1.30 (1.10–1.54)	0.002	1.30 (1.1–1.53)	< 0.001	1.36 (1.18–1.56)
**City of residence**												
*São Paulo*		1.00		1.00		1.00		1.00				
*Others*	0.186	0.91 (0.78–1.05)	0.187	0.91 (0.78–1.05)	0.192	0.91 (0.78–1.05)	0.214	0.91 (0.79–1.06)	–	–	–	–
**Graduation School**												
*Private*		1.00		1.00		1.00		1.00		1.00		1.00
*Public*	0.086	1.13 (0.98–1.30)	0.079	1.13 (0.99–1.30)	0.087	1.13 (0.98–1.29)	0.089	1.13 (0.98–1.29)	0.103	1.12 (0.98–1.29)	0.089	1.13 (0.98–1.29)
**Local of Graduation**												
*São Paulo*												
*Others*	0.432	1.07 (0.91–1.26)	0.427	1.07 (0.91–1.26)	0.426	1.07 (0.91–1.26)	–	–	–	–	–	–
Medical Residency **												
*No*	1.00		1.00		1.00		1.00		1.00		1.00	
*Yes*	< 0.001	1.69 (1.39–2.03)	< 0.001	1.69 (1.39–2.03)	< 0.001	1.69 (1.39–2.03)	< 0.001	1.69 (1.39–2.03)	< 0.001	1.69 (1.39–2.03)	< 0.001	1.69 (1.40–2.04)
Specialist***												
*Non specialist*		1.00										
*Specialist*	0.769	0.97 (0.78–1.20)	–	–	–	–	–	–	–	–	–	–

^*^ *p*-value calculated by the method of Wald

^**^Monthly wage adjusted by the Big Mac Index

^**^Conclusion of medical residency up until 3 years after the end of the contract

^***^Specialty at the time of employment

## Discussion

### Top of form

The present study strongly highlights the high turnover of physicians in PHC services in the East Zone of São Paulo. The average time until the termination of the contract was 2.14 years, that is, the professionals remained around 2 years in the services, although some individuals who stayed longer or very short periods in the PHC services influence this average. Almost half of the professionals left PHC shortly after admission (1.17 year). In other words, after hiring a medical professional, the probability of termination of his contract in the first year is 45%. In 5 years, this probability rises to almost 90%. Thus, only 10% of all active medical workforce remained in the services after 5 years of study follow-up.

Among the individual characteristics of the physicians studied here, the homogeneity of the gender variable was noted, with a slight female predominance: 52.6% of the PHC physicians at the follow-up were women. Though alone this data does not reflect the phenomenon of feminization of medicine, the Brazilian medical literature has already reported this process [35].

Regarding the age of doctors at the time of hiring, the group studied is quite young: 55.2% were under 30 years old. Although the profession’s youth in Brazil is a trend, in the total population of doctors in the country, in this same age group (up to 29 years) are 42.6% of professionals [35]. Brazil has witnessed in the last decade the opening of many new private medical schools, including the city of São Paulo, one of them Santa Marcelina, The increased supply of newly graduated doctors, combined with the large availability of vacancies in the municipality’s PHC, could lead to an even younger profile of the occupants of these jobs. Direct entry into the labor market, either because of difficulty in accessing a specialty residency, or the desire for more immediate financial gains (need to pay off public or private educational debts assumed at undergraduate level), is a phenomenon that needs to improve considering its possible impact not only on the longevity in PHC services, but also on the quality of care provided to the population [35–42],

Younger individuals, ≤25 years, averaged 1.4 years in PHC services, while those aged 30 and over remained for 2.7 years. However, when adjusting the final model, age was not significant as a disengagement factor. Collinear to age, the time since graduation was relevant, with statistical significance, in the dismissal of doctors, and those graduated less than 2 years spent less time in PHC services, with a mean of 1.7 years.

Most of the physicians (78%) had a 40-h workweek, a workload that reached 89% of the FHS doctors. The workload, as shown, is a contributing factor to the TEC. Doctors on a 40-h weekly basis had a significantly higher occurrence of TEC. Previously exclusively set at 40 h per week by federal determination [48], the journey of doctors in the PHC and FHS gained other possible configurations over the years, being allowed a workload of 20 or 30 h per week as of 2011 [49].

As with the full 40-h workday, the higher starting salary remained significant after adjustment as a factor of lower severance. Two variables, the 40-h workday and the highest salary, are related, since salaries are largely proportional to the hours hired or worked.

PHC Santa Marcelina has already adopted a salary additions policy for districts in the East Zone that have greater difficulty in attracting doctors, such as Itaim, Guaianases and Tiradentes, due to the great distance of these regions to the city center. The practice of different wages within the same institution is a sensitive theme that runs into labor laws and can lead to greater wage inequalities between doctors and other professionals equally essential to PHC [50]. It has been shown that salary is a factor of attraction in PHC [23] but is insufficient to guarantee its permanence in medium and long term [22].

It was found in the study that the vast majority of doctors (88.1%), upon joining the PHC service, had no medical specialty, i.e. had not completed a residency program or obtained the title of specialist by the Medical Society. There is usually no requirement for any specialty title to occupy the position of physician in PHC services. Job selection and hiring processes refer to “general practitioners”, understood as the professional with a general medical background, i.e., the doctor with a completed degree and registration in the regional medical council.

The lack of specialization at the time of admission could be explained by the low average age of the physicians in this study. That is, for many, there was still not enough time to complete the specialization. It is also known that there are not enough Medical Residency positions for all medical graduates; paradoxically, there is a low demand for Family and Community Medicine Residency vacancies, a specialty that has insufficient specialists to supply the jobs of doctors at PHC. Some municipalities in southern Brazil, such as Florianopolis, exception to the rule, insert in the contracting notices for FHS, the residency criterion or specialist title, aiming at a higher qualification of PHC human resources [51].

Among the findings of the study, 11.5% of physicians (who were not specialists at the time of admission) completed a residency within 3 years after the termination of contract in the PHC Santa Marcelina. This was a factor of strong impact on TEC, indicating that for this group of physicians, work in PHC may have been merely a transition between the end of graduation and beginning of residency, often in non-PHC specialties.

The percentage of specialists among admission (only 12%) increased significantly among those who were discharged; 45% of physicians who left PHC services throughout the study had a specialist title until 2016, the last year of follow-up. After leaving PHC, they specialized mainly in Gynecology and Obstetrics (13.2%), Pediatrics (12.9%), Family and Community Medicine (12.4%) and Clinical Medicine (11.0%). Although specialties are all suitable and relevant to PHC, it is noteworthy that Family and Community Medicine was not the priority choice.

Therefore, it should be a matter of concern for the human resources policies in PHC the entry of large numbers of young doctors, recent graduates, without specialization and with post-retirement professional choices unrelated to work and PHC goals.

Although the number of doctors in Brazil has been growing exponentially, increasing 3.5 times more than the population between 1980 and 2010 [52], the persistent increase in the number of doctors did not benefit homogeneously all Brazilian citizens [42]. The city of São Paulo had 59,934 doctors [35] in professional activity in 2018, registered with the Regional Council of Medicine (CRM), a ratio of 4.98 doctors per 1000 inhabitants, compared to capitals with the highest concentrations of doctors in the world and our study showed lack of these professionals in the periphery of the same city. The “*Mais Médicos”* federal Program (More Doctors) created in 2013 [53], and its successor, in 2019, the “*Médicos pelo Brasil”* (Doctors for Brazil) Program [54] have given rise to important policy debates but still haven’t resulted in a full coverage of PHC.

The findings of the present study point to concrete obstacles to the expansion of PHC, whose proportion of population coverage is insufficient, even in the city of São Paulo, since it does not reach 50% in the region studied. Even when overcome, the difficulty of hiring doctors in PHC is compounded by the high turnover of those who enter the services. Leaving after a short-term stay constantly deflects FHS teams or leaves open jobs at UBSs, a phenomenon that requires extra efforts and replacement costs that are not always successful.

Some of the attributes of PHC are compromised with this dynamic, such as the ability to solve the vast majority of the population’s health problems and continuousness, which presupposes continuity of care, building bonds and accountability between professionals and users over time, permanently and consistently.

Studies have also shown that high staff turnover at PHC has a financial impact that includes the actual cost of entering and leaving staff, the cost of temporary replacements, as well as the cost of training, immersion activities, and alignment with care lines and the organization of services [55]. Overworking remaining professionals and breaking ties among the PHC team are other possible effects of physician turnover. On the population side, as the services most affected are those in regions of greatest social vulnerability, the vicious cycle of the lowest care associated with the worst health indicators seems to support a feedback system [56].

The methodological choice of physician dismissal measurement through the survival curve allowed the use of all study participants, regardless of the date of entry. Such methodology has been used in other countries [57–60] and in Australia, survival curves are essential as a methodology to track the effectiveness of national health manpower policies [58]. Retention in both cases (before and after retention policies) was higher than that observed in this study. Russell et al. [59] were able to demonstrate, through survival analysis techniques, the influence of factors associated with geographic location and population size on the retention phenomenon of physicians.

In Brazil, literature from the last decade has highlighted the inequalities in the supply and distribution of doctors, as well as the official policies for the establishment and provision of doctors implemented throughout the history of the health system. The high turnover of physicians in PHC services in Brazil is associated with multiple factors that generate professional dissatisfaction, such as inadequate working conditions, excessive workload, low pay and lack of job, career and salary plans [61–65]. The reasons that may favor the permanence are the identification with the PHC, the professional vocation and the perspective of serving the community [25, 37, 44, 51].

Resistance factors to exclusive dedication or permanence in a single workplace in PHC must also be considered as the characteristics of the medical profession in Brazil, are defined by the double public and private practice [45] and by multiplicity of work relationships (almost half of doctors have three or more jobs), long working hours (two thirds of doctors work more than 40 h per week), working shifts (45% make at least one shift per week), beyond the prospect of higher incomes that is usually only achieved through accumulation of jobs and activities. According to the Medical Demography in Brazil [66], the average physician’s workload is more than 50 h per week, and almost a third of professionals work more than 60 h per week. Doctors work, on average, in three different jobs, with more than 30% accumulating four or more contracts. The professional usually occupies more than one job in UHS, often hired by more than one employer in the same municipal network. Most doctors working in the public sector share their work schedule with a private practice or practice in the private sector. It should be considered that in the city of São Paulo there is a large and diverse private network that employs part of the doctors who simultaneously work for the UHS [45, 66].

Throughout the history of the Brazilian health system, many governments have attempted to ensure the supply of doctors in PHC and other levels of care [67–70], including the Rondon Project, the Internationalization of Health Work (PITS), the Program to Support the Training of Specialist Physicians in Strategic Areas (Pro-Residency), the Appreciation Program for Primary Care Professionals (PROVAB). The two most recent federal medical provision programs, “*Mais Médicos*” and “*Médicos pelo Brasil*” [53, 54], did not use in their conceptions and legal frameworks evidence on physician turnover and permanence in PHC, but both set criteria for municipalities’ eligibility that would benefit from the programs [39–54, 66].

A strength of the study was the possibility of the integration of the human resources department’s administrative database of the medical contracting organization in PHC with the most comprehensive study database, Medical Demography in Brazil, which allowed for an unprecedented approach to information and characteristics of physicians not reached by the isolated databases. This permitted the use of a large number of physicians over a long time period. However, this study has limitations, the main one being the limit range of findings, restricted to the available data and the variables selected for analysis, and the use of secondary data only, always subject to incompleteness of information and lack of systematization of records such as the authorization of doing less than 40 h/week was approved in 2011, 5 years after the study beginning that could be a confounding factor not measured thoroughly. This study could be extended to all São Paulo PHC and could be an interesting observatory for prospective cohort. A qualitative study could amplify the range of variables.

The turnover of doctors in PHC, is multifactorial. Various individual characteristics of physicians, service administrators and employers, as well as geographical, professional, financial and educational factors, are associated with retention of physicians in PHC [71–76]. Higher turnover has also been related to job dissatisfaction, conflicts between work and family care [77], as well as stress and burnout in a professional setting [78, 79].

Therefore, there are several research fronts and quantitative and qualitative methodologies that need to be considered for the systemic and complete approach to the problem. In the case studied, information related to physicians’ personal choices, perception of security and violence in the East Zone, and opinion about the management institution’s profile and conduct, just to name three examples of topics not reached by secondary data, would be relevant to a better understanding of physicians’ withdrawal and permanence factors in PHC services.

## Conclusions

PHC in UHS in the East Zone of the city of São Paulo has been occupied by a medical workforce with a majority profile of young graduates, without specialization, with little or no previous professional experience, who remain for a short time or leave early on the services.

The study shows the relevance of characteristics of the work contract (part-time work less than 40 h per week and higher initial salaries), individual characteristics (the fact that the doctor has a short time since graduation) and personal or professional choices (the termination of employment to attend medical residency).

Strategies within human resources policies to further attract and retain physicians must be combined and multiple, capable of acting on both modifiable factors at the time of job posting and hiring (eg. Salary, working hours and working conditions) and by less modifiable factors by employers upon admission (personal determination to study a certain medical specialty, for example).

## Supplementary Information

**Additional file 1.**

## Data Availability

The datasets generated and/or analyzed during the current study are not publicly available due to ethical issues related to participant confidentiality imposed by the Ethics Committee of the medical School of the University of São Paulo. Data from this paper are available upon request to the Ethics Committee of the medical School of the University of São Paulo. Mailing address: 251 Dr. Arnaldo Avenue- Cerqueira César – 01246-000 – São Paulo – SP – Brazil. Phone: + 55 (11) 3893–4401: Dr. Maria Aparecida Azevedo Koike Folgueira.

## Abbreviations

95%CI: 95% confidence intervals
AMB: Brazilian Medical Association
BMI: Big Mac Index
CNRM: National Commission of Medical Residency
CRM: Medical Regional Councils
TEC: Early termination of contract
FHC: Family Health Care program
FHS: Family Health Strategy
HDI: Human development index
HR: Hazard Ratio
LDB: Law of Directions and Bases for Education
PHC: Primary health care
SPSS: Statistical Package for the Social Sciences
UHS: Unified Health System
THU: Traditional Health Care Unit
