# Supplementary material for: Physician turnover in primary health care services in the East Zone of São Paulo City, Brazil: incidence and associated factors
Source: BMC Health Serv Res. 2022 Feb 4;22:147. doi: 10.1186/s12913-022-07517-1 (PMC8815273; doi:10.1186/s12913-022-07517-1)
Supplement: Supplementary file 1 — Additional file 1. [file 12913_2022_7517_MOESM1_ESM.docx]

Supplementary Material

“Physician Turnover in Primary Health Care Services in the East Zone of São Paulo City, Brazil: Incidence and associated factors”. Bourget, Cassenote and Scheffer, 2021.

List of variables used in the study:

1. Work time: difference between the date of initial employment and the date of the physician’s termination of contract from PHC up until the date of the closing of the database in December 31st, 2016. This is the time until the early termination of contract, the outcome of the survival analysis;
2. Termination of contract (TEC): situation of the physician’s employment contract within the institution. In this study, all contract terminations were motivated by the request of the physicians themselves. Any dismissals or terminations made by the employer were not considered status in this study. This is the state variable of the survival analysis;
3. Gender: physician gender;
4. Age: physician´s age at the beginning of the contract of the PHC studied;
5. Year of Initial Employment Date: year in which the physician started to be part of the human resources of the institution;
6. Weekly workload: workload relative to the weekly journey of the physician´s work in PHC. Variable categorized according to full workload (8 hours a day, 5 days a week) and partial (whatever workload fewer than 40 hours a week);
7. Place of work: physician´s workplace in PHC: either Family Health Strategy (FHS) or Traditional Health Care Unit (THU);
8. Initial salary at employment date: Initial Salary at the start of employment in PHC. Adjusted by Big Mac Index (BMI), indicative index of purchasing power;
9. Time frame since graduation at the time of Initial employment: time calculated between the year of graduation and the year of initial employment in PHC. Variable categorized to identify recent graduates (2 years or less);
10. City of residency – where physician resides: City associated to the postal code of physician´s home address informed in the professional registry on admission in PHC (City of São Paulo or Other municipalities);
11. Type of graduation school: public or private; nature of the physician´s graduation course/school. The reference was based on the Law of Directions and Bases for Education (LDB of 1996) which defines two administrative categories of teaching institutions: public are the ones that are created or incorporated, maintained and managed by the public power and the private are “maintained and administrated by physical persons or of private rights”;
12. City of graduation: municipality where the physician´s school/course is localized;
13. Specialty on initial employment date: registered physician specialty on moment of Initial Employment Date in PHC. Variable categorized considering “specialist” physician that has concluded Medical Residency or possesses a Specialist title issued by the Specialist Society filiated to Brazilian Medical Association; and “non specialist” the physician without title in the categories cited above;
14. Specialty at the present moment: physician specialty at the present moment (2016, last year updated of the bases used). Variable categorized considering “specialist” physician that concluded medical residency or possesses a specialist title issued by the specialist society filiated to Brazilian Medical Association; and “non specialist” the physician without title in the categories above;
15. Medical residency after employment: conclusion of Medical residency posteriorly (up to 3 years) from the date of the physician´s termination of contract from PHC.
